# The association between early menarche and higher-risk cardiometabolic profile: a dose–response analysis of the Tabari cohort at enrollment phase

**DOI:** 10.3389/fcvm.2023.1241179

**Published:** 2023-09-01

**Authors:** Motahareh Kheradmand, Zeinab Hamzehgardeshi, Zohreh Shahhosseini, Razie Mirjalili, Mahmood Moosazadeh

**Affiliations:** ^1^ Health Sciences Research Center, Addiction Institute, Mazandaran University of Medical Sciences, Sari, Iran; ^2^ Professor, Department of Reproductive Health and Midwifery, Sexual and Reproductive Health Research Center, Mazandaran University of Medical Sciences, Sari, Iran; ^3^ Professor of Reproductive Health, Sexual and Reproductive Health Research Center, Department of Reproductive Health and Midwifery, Faculty of Nursing and Midwifery, Mazandaran University of Medical Sciences, Sari, Iran; ^4^ Student Research Committee, Faculty of Medicine, Mazandaran University of Medical Science, Sari, Iran; ^5^ Gastrointestitional Cancer Research Center, Non-Communicable Disease Institute, Mazandaran University of Medical Sciences, Sari, Iran

**Keywords:** age at menarche, diabetes, hypertension, waist circumference, BMI

## Abstract

**Objectives:**

The association between age at menarche and higher-risk cardiometabolic factors is controversial and more strands of evidence are required. Therefore, in this study, we aimed to investigate the effect of early menarche on cardiometabolic profile in a large-scale cohort population.

**Study design:**

Data collected in the enrollment phase of the Tabari cohort study were utilized for the present study. We analyzed data from 6,103 women aged 35–70 years. Logistic regression and dose–response (trend) analyses were used to investigate the effect of early menarche on prevalence of diabetes, dyslipidemia, obesity, high waist circumference (WC), high waist-to-hip ratio (WHR), and high waist-to-height ratio (WHtR).

**Results:**

The results of the adjusted logistic regression analysis showed that women who experienced early menarche had significantly higher odds of obesity (odds ratio: 1.64, 95% CI: 1.36–1.99, *P* for trend <0.001), high WC (odds ratio: 1.34, 95% CI: 1.07–1.67, *P* for trend = 0.035), high WHR (odds ratio: 1.32, 95% CI: 1.05–1.66, *P* for trend = 0.057), and high WHtR (odds ratio: 1.83, 95% CI: 1.22–2.74, *P* for trend = 0.006) compared to those aged ≥14 at menarche. The prevalence of dyslipidemia was also higher among women who experienced early menarche than in women aged ≥14 at menarche (79.9% vs. 76.6%), but the difference was not statistically significant (*P* = 0.098). Additionally, each year of earlier menarche was significantly associated with an increase in the chance of diabetes (by 5%), obesity (10%), high WC (5%), and high WHtR (13%).

**Conclusion:**

The present study showed that early menarche is a strong predictor for later development of obesity and diabetes, and for high WC, WHR, and WHtR. Among all factors examined, age at menarche had the greatest predictive power for WHtR. As an age-dependent anthropometric index for central obesity, WHtR is more suitable as an index for identification of individuals with increased cardiometabolic risk.

## 1. Introduction

Cardiovascular and metabolic diseases are major leading causes of death worldwide (1). During the last decade, studies have shown that manipulating the modifiable risk factors for cardiovascular disease (CVD), such as obesity, smoking, and dyslipidemia, can reduce CVD mortality, especially in developed countries (2). Nevertheless, beyond the conventional risk factors, women face additional sex-specific risk factors, such as early/late menarche, polycystic ovary syndrome, infertility, adverse pregnancy outcomes, gestational diabetes, and breastfeeding. All the above-mentioned factors increase the future risk of cardiovascular disease (2, 3). Age at menarche is defined as age at the first menstrual period, which is a sign of reproductive puberty. It has been reported that age at menarche is related to health outcomes in later life (4). The association between age at menarche and various outcomes has been investigated in the literature. This variable has been reported to be associated with risk of cardiovascular death (5), coronary heart and cerebrovascular diseases (6), depression and antisocial behavior in adulthood (7), and endometrial cancer (8).

Moreover, several studies have reported controversial results on the association between age at menarche and various cardiometabolic risk factors (6, 9–11). However, these relationships can be significantly affected by many factors such as ethnicity, geographical region, and socioeconomic status (12). Therefore, we aimed to investigate the association of age at menarche with lipid profile, anthropometric indices, and several other clinical risk factors for cardiovascular disease in a large-scale, population-based cohort study.

## 2. Methods

### 2.1. Study participants

In the present cross-sectional study, we utilized data collected in the enrollment phase of the Tabari cohort study (TCS). The TCS is a component of a national cohort study named “Prospective Epidemiological Research Studies in IrAN” (PERSIAN). The TCS was conducted in Sari Township, Mazandaran Province (in the north of Iran and in the vicinity of the Caspian Sea). In total, 10,255 participants aged 35–70 years (7,012 and 3,243 individuals from urban and mountainous regions, respectively) were recruited during the enrollment phase of the TCS, of whom 6,106 were women. Due to a lack of blood samples, three participants were excluded; ultimately, 6,103 participants were included in the final analysis. The rationale, objectives, data collection methods, and design of the PERSIAN cohort and the TCS have been reported in previously published articles (13–15). Briefly, data collection methods consisted of questionnaires, anthropometric measurements, and blood pressure tests. In addition, blood and urine samples were collected from all participants.

### 2.2. Variables analyzed in the present study

For the present study, we analyzed the following variables collected during the enrollment phase of the TCS: age, height, weight, waist circumference (WC), hip circumference, socioeconomic status, residency, education level, number of pregnancies, age at menarche, menopause (yes/no), systolic and diastolic blood pressure, history of hypertension and diabetes, and levels of fasting blood glucose (FBS), total cholesterol (TC), triglycerides (TGs), high-density lipoprotein (HDL), and low-density lipoprotein (LDL).

### 2.3. Blood sample collection

All participants in the study were instructed to refrain from eating and drinking (except water) for 8–12 h before sample collection. To minimize the effect of fluctuations in clinical and laboratory parameters, all sampling was conducted between 7 and 9 in the morning. In total, 25 ml of blood was collected from each participant in the TCS by expert staff members. After blood sample collection, the serum was separated and the samples were stored in a biobank for use during the follow-up period: 0.5 ml of serum was used for biochemical tests, and FBS, TC, TG, and HDL levels were subsequently measured using the BT 1500 (Biotechnica, Italy).

### 2.4. Anthropometric indices

All anthropometric indices, including weight, height, body mass index (BMI), waist circumference (WC), waist-to-hip ratio (WHR), and waist-to-height ratio (WHtR) were measured by trained staff according to standardized methodologies (16). For the present study, participants were categorized as follows: participants with BMI ≥30 were classified as obese, and participants with WC ≥88 (17), WHR ≥0.85 (18), and WHtR ≥0.5 were classified into abnormal groups on the corresponding variables (19).

### 2.5. Age at menarche

Age at menarche was recorded in the TCS database as a quantitative, discrete variable. The onset mean age of menarche, as well as its classification, varies among people of different ethnicities. In the present study, we categorized all participants into three groups: early menarche (at age 11 or younger), menarche at age 12–13, or menarche at age ≥14 (1).

### 2.6. Statistical analysis

Data were analyzed using SPSS version 26 (IBM SPSS Corp, SPSS Statistics ver. 26, USA). The chi-squared test was conducted to compare categorized demographical and clinical variables between age-at-menarche groups (early, aged 12–13, and aged ≥14 years). Quantitative variables were compared using independent *t*-tests. The relationships of age at menarche with lipid profile, glucose level, and anthropometric indices were assessed using Pearson's correlation coefficients. Crude and adjusted logistic regression models were constructed to estimate the odds of diabetes, hypertension, dyslipidemia, obesity, high WC, high WHR, and high WHtR in women with different menarche ages, adjusting for potential confounding variables such as age, number of pregnancies, area of residence, education level, and menopause. Additionally, for the dose–response analysis, we estimated *P* for the trend according to the logistic regression model (20).

Trend analysis (taking the quantitative variable of age at menarche as an independent variable) was carried out to determine the effect of age at menarche on cardiometabolic outcomes such as diabetes, hypertension, dyslipidemia, obesity, WC, WHR, and WHtR using an adjusted logistic regression model.

In the present study, we defined hypertension as systolic blood pressure ≥140 mm Hg, diastolic blood pressure ≥90 mm Hg, a history of hypertension diagnosis, or taking antihypertensive medications. Diabetes mellitus was defined as fasting blood sugar ≥126 mg/dl, a history of diagnosis, or taking glucose-lowering medications. Dyslipidemia was defined as TC ≥200 mg/dl, TG ≥150 mg/dl, LDL-C ≥130 mg/dl, or HDL-C <50 (21).

## 3. Results

This study analyzed data from 6,103 women aged 35–70 years (areas of residence: 66.6% urban and 33.4% mountainous). Most participants were in the age group of 40–49 years at the time of enrollment. The prevalence rates of diabetes, hypertension, dyslipidemia, high WC, high WHR, high WHtR, and obesity were 18.3%, 24.9%, 77.6%, 69.6%, 73.5%, 91.4%, and 42.4%, respectively. In total, 65.5% of women had had more than three pregnancies and 29% had had more than five pregnancies. At the time of enrollment, 45.1% of participants had experienced the menopause.

The mean ± SD age at menarche among the study participants was 13.66 ± 1.70 years. The median (first and third quartile) age at menarche was 14 years (13–15), with the minimum and maximum ages being 9 and 22 years, respectively. The prevalence rates of early menarche, menarche at 12–13 years, and menarche at ≥14 years of age were 8.7%, 39%, and 52.4%, respectively. The mean ± SD age at menarche of obese women (13.50 ± 1.74) was significantly lower than that of non-obese women (13.78 ± 1.66) (*P* < 0.001).

Among women who experienced early menarche, menarche at 12–13 years, and menarche at ≥14 years of age, the proportions living in urban regions were 78.6% (415 of 528), 74.6% (1,774 of 2,379), and 58.7% (1,877 of 3,196), respectively. It should be noted that the rate of early menarche among the 2,037 participants living in mountainous regions was 5.5% (113 women), while the rate among the 4,066 participants living in urban regions was 10.2% (415 women). This difference was statistically significant (*P* < 0.001).

The prevalence rates of hypertension in women who experienced early menarche, menarche at 12–13 years, and menarche at ≥14 years of age were 22.7% (120 of 528), 24.5% (584 of 2,379), and 25.6% (818 of 3,196), respectively (*P* = 0.315). The prevalence rate of diabetes in each of the three age-at-menarche groups were 17% (90 of 528), 18.7% (444 of 2,379), and 18.3% (584 of 3,196), respectively (*P* = 0.682), and the rates of obesity were 52.7% (278 of 528), 44.7% (1,064 of 2,379), and 38.9% (1,244 of 3,196), respectively (*P* < 0.001).

The prevalence of dyslipidemia was higher in women who experienced early menarche than in women who experienced menarche at 14 years of age or older. However, this difference was not statistically significant (79.9% vs. 76.6%, respectively, *P* = 0.098). In contrast, high WC and high WHtR were significantly more common in women who experienced early menarche than in those who experienced menarche at ≥14 years of age (*P* = 0.022 and *P* = 0.002, respectively) (Table 1).

**Table 1:** Demographic, anthropometric, and clinical characteristics of the study participants, grouped by age at menarche.

Variables		Age at menarche			*P*-value (chi-square test)
		≤11	12–13	≥14	
Age group	35–39	101 (19.1)	427 (17.9)	498 (15.6)	<0.001
	40–49	209 (39.6)	855 (35.9)	1,085 (33.9)	
	50–59	142 (26.9)	723 (30.4)	1,004 (31.4)	
	60–70	76 (14.4)	374 (15.7)	609 (19.1)	
Area of residence	Urban	415 (78.6)	1,774 (74.6)	1,877 (58.7)	<0.001
	Mountainous	113 (21.4)	605 (25.4)	1,319 (41.3)	
Socioeconomic status	1 (poorest)	83 (15.7)	420 (17.7)	892 (27.9)	<0.001
	2	103 (19.5)	461 (19.4)	691 (21.6)	
	3	121 (22.9)	478 (20.1)	636 (19.9)	
	4	122 (23.1)	489 (20.6)	525 (16.4)	
	5 (richest)	99 (18.8)	531 (22.3)	452 (14.1)	
Marital status	Single	69 (13.1)	299 (12.6)	403 (12.6)	0.951
	Married	459 (86.9)	2,080 (87.4)	2,793 (87.4)	
Education level	University/college	85 (16.1)	470 (19.8)	486 (15.2)	<0.001
	9–12 years	170 (32.2)	740 (31.1)	658 (20.6)	
	6–8 years	70 (13.3)	278 (11.7)	270 (8.4)	
	1–5 years	142 (26.9)	502 (21.1)	1,013 (31.7)	
	No schooling	61 (11.6)	389 (16.4)	769 (24.1)	
Menopause	No	318 (60.2)	1,334 (56.1)	1,699 (53.2)	0.004
	Yes	210 (39.8)	1,045 (43.9)	1,497 (46.8)	
Number of pregnancies	0	34 (6.4)	130 (5.5)	200 (6.3)	<0.001
	1	36 (6.8)	139 (5.8)	194 (6.1)	
	2	130 (24.6)	576 (24.2)	664 (20.8)	
	3	119 (22.5)	545 (22.9)	642 (20.1)	
	4	81 (15.3)	364 (15.3)	480 (15)	
	≥5	128 (24.2)	625 (26.3)	1,016 (31.8)	
Hypertension	No	408 (77.3)	1,795 (75.5)	2,378 (74.4)	0.315
	Yes	120 (22.7)	584 (24.5)	818 (25.6)	
Diabetes	No	438 (83)	1,935 (81.3)	2,612 (81.7)	0.682
	Yes	90 (17)	444 (18.7)	584 (18.3)	
Waist circumference (cm)	<88	133 (25.2)	727 (30.6)	995 (31.1)	0.022
	≥88	395 (74.8)	1,652 (69.4)	2,201 (68.9)	
WHR	<0.85	127 (24.1)	671 (28.2)	821 (25.7)	0.044
	≥0.85	401 (75.9)	1,708 (71.8)	2,375 (74.3)	
WHtR	<0.5	29 (5.5)	189 (7.9)	309 (9.7)	0.002
	≥0.5	499 (94.5)	2,190 (92.1)	2,887 (90.3)	
BMI	<30	250 (47.3)	1,315 (55.3)	1,952 (61.1)	<0.001
	≥30	278 (52.7)	1,064 (44.7)	1,244 (38.9)	
Dyslipidemia	No	106 (20.1)	511 (21.5)	748 (23.4)	0.098
	Yes	422 (79.9)	1,868 (78.5)	2,448 (76.6)	

WHR, waist-to-hip ratio; WHtR, waist-to-height ratio; BMI, body mass index.

The results of the crude and adjusted logistic regression models are presented in Table 2. After adjustment for potential confounding variables, such as age, number of pregnancies, menopause, socioeconomic status, area of residency, and education level, significantly higher odds ratios for obesity (1.64; 95% CI: [1.36, 1.99], *P* for trend <0.001), high WC (1.34; 95% CI: [1.07, 1.67], *P* for trend = 0.035), high WHR (1.32; 95% CI: [1.05, 1.66], *P* for trend = 0.057), and high WHtR (1.83; 95% CI: [1.22, 2.74], *P* for trend = 0.006) were observed for women who experienced early menarche than for those who experienced menarche at ≥14 years of age. Additionally, based on trend analysis, each year of earlier menarche was significantly associated with an increase in the prevalence of diabetes (by 5%), obesity (by 10%), high WC (by 5%), and high WHtR (by 13%).

**Table 2:** Association between early menarche and cardiometabolic profile according to crude and adjusted logistic regression models.

Variables	Crude model		Adjusted model*		*β* (trend analysis, adjusted model)*	*P* for trend
	≤11	12–13	≤11	12–13		
Diabetes (yes)	0.92 (0.72, 1.17	1.03 (0.89, 1.18)	1.06 (0.82, 1.38)	1.15 (0.99, 1.33)	−0.05**	0.177
Hypertension (yes)	0.85 (0.69, 1.06)	0.95 (0.84, 1.07)	0.98 (0.78, 1.24)	1.04 (0.91, 1.19)	−0.01	0.823
Dyslipidemia (yes)	1.22 (0.97, 1.53)	1.12 (0.98, 1.27)	1.22 (0.97, 1.54)	1.11 (0.97, 1.27)	−0.02	0.117
BMI ≥30	1.74 (1.45, 2.10)**	1.27 (1.14, 1.41)**	1.64 (1.36, 1.99)**	1.22 (1.09, 1.37)**	−0.10**	<0.001
Waist circumference ≥88 (cm)	1.34 (1.09, 1.66)**	1.03 (0.92, 1.15)	1.34 (1.07, 1.67)**	1.03 (0.91, 1.17)	−0.05**	0.035
WHR ≥0.85	1.09 (0.88, 1.35)	0.88 (0.78, 0.99)	1.32 (1.05, 1.66)**	1.03 (0.90, 1.17)	−0.03	0.057
WHtR ≥0.5	1.84 (1.24, 2.73)**	1.24 (1.03, 1.50)**	1.83 (1.22, 2.74)**	1.22 (0.99, 1.49)	−0.13**	0.006

WHR, waist-to-hip ratio; WHtR, waist-to-height ratio; BMI, body mass index.

Reference group for age at menarche: ≥14 years.

* Adjusted for the variables: age, area of residence, socioeconomic status, education level, number of pregnancies, and menopause.

** *P*-value < 0.05.

**Figure 1:**
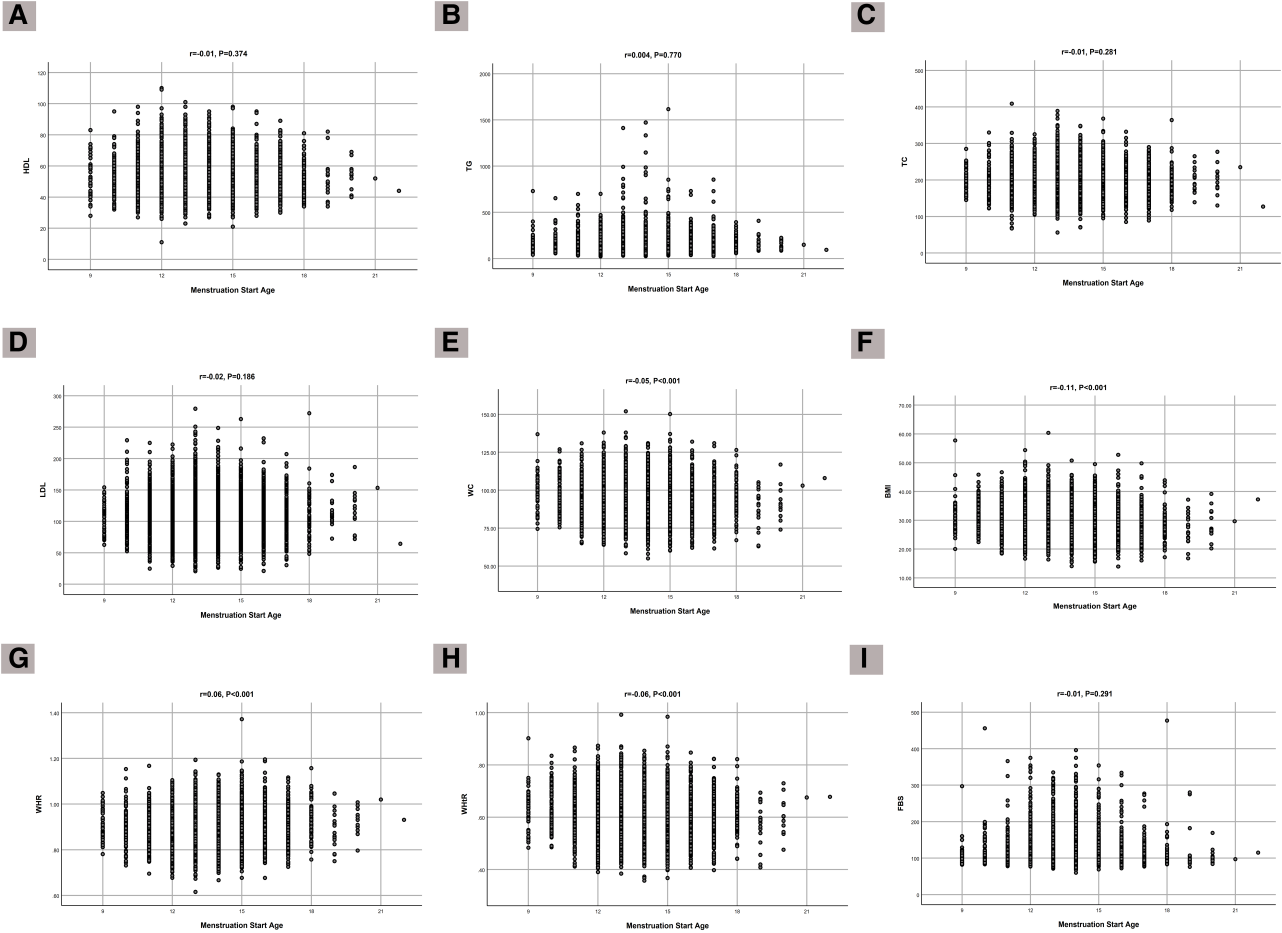
Correlation of age at menarche with (**A**) HDL, (**B**) TG, (**C**) TC, (**D**) LDL, (**E**) FBS, (**F**) WC, (**G**) BMI, (**H**) WHR, and (**I**) WHtR.

The correlations of age at menarche with lipid profile, glucose level, and anthropometric indices are shown in Figure 1. There were significant correlations with BMI (*r* = −0.11, *P* < 0.001), WHR (*r* = 0.06, *P* < 0.001), WC (*r* = −0.05, *P* < 0.001), and WHtR (*r* = −0.06, *P* < 0.001).

## 4. Discussion

The present study investigated the association between early menarche and cardiometabolic profile. The results of adjusted logistic regression models showed significantly higher odds of obesity (64%), high WC (34%), high WHR (32%), and high WHtR (83%) for women who experienced early menarche compared to those who experienced menarche after 14 years of age. The results of trend analysis showed that one-year lower menarche age was inversely associated with the chance of developing obesity and diabetes and of having a high WC and WHtR.

The mean age at menarche among TCS participants (13.66 ± 1.70 years old) was older than that reported in the Southwest Iran Hoveyzeh Cohort Study population (12.60 ± 1.76 years old) (22) and that reported in a systematic review of Iranian girls (12.81 years) (23), as well as the mean age at menarche in Canada (12.72 ± 1.05) (24). In contrast, Korean and Chinese women have been found to be older at menarche than our study participants (2, 4). The mean age at menarche in a large UK cohort study was slightly lower than that observed in the present study (6). The wide range of ages at menarche across various geographical areas, even within a given country, emphasizes the effects of ethnicity and climate on the onset of the menstrual cycle (25). It has been reported that climate change can influence the onset of menarche, via the associated increase in levels of toxins, levels of water and soil pollutants, and famine (26). Consistent with previous research, the findings of our study showed a higher prevalence of early menarche in urban areas of residence.

In our study, the prevalence rates of diabetes and hypertension did not differ in women who experienced menarche at different ages. However, the rates of obesity and dyslipidemia varied significantly with age at menarche. A study carried out with Korean women reported a higher prevalence of hypertension, diabetes, metabolic syndrome, and high WC in women who experienced early menarche; however, the definition of early menarche was different from that applied in the current study (2). The findings of the present study strongly agree with those of previous studies regarding the association between early menarche and obesity (27).

Regarding anthropometric indices, our findings showed that the prevalence of abnormal values on all anthropometric indices, including high WC, high WHR, high WHtR, and obese BMI, was higher among women who experienced early menarche than among those who experienced menarche at ≥14 years of age. The findings of this study, in agreement with other studies, suggest that early menarche is a triggering factor for accumulation of fat mass, which can act as a neuroendocrine stimulant for menstruation. On the other hand, early exposure to gonadal steroids can lead to abdominal obesity in late adulthood (28–30).

The results of the dose–response analysis showed that each year of earlier menarche was associated with an increase in the chance of obesity and diabetes and of high WC and WHtR. The results of a study conducted with a Chinese population showed that earlier onset of menarche by 1 year was associated with higher odds of hypertension (31). An association between early menarche and an increased prevalence of diabetes has also been reported in a systematic review (32).

One of the unique features of the present study is that the associations of age at menarche with all anthropometric indices were investigated; in particular, we analyzed WHtR, which is a less age-dependent variable. In addition, lipid profiles and common noncommunicable diseases were also investigated in this study.

One of the limitations of the present study is the influence of recall bias on the accuracy of reporting of age at menarche, given that menarche had occurred a long time ago for the participants and had to be recalled by them for self-report.

In conclusion, the results of this study suggest that early menarche is a strong predictor of obesity, diabetes, and elevated measures on some anthropometric indices. Therefore, early menarche can be used to identify women who are at greater risk of cardiometabolic diseases and as a valuable measure for preventive strategies.

## Data Availability

The original contributions presented in the study are included in the article/Supplementary Material; further inquiries can be directed to the corresponding author.
